# A study on the influence of physical education teachers’ teaching beliefs and attributions of teaching success and failure on their effective teaching behaviors

**DOI:** 10.1371/journal.pone.0321137

**Published:** 2025-04-11

**Authors:** Ye Wei

**Affiliations:** School of Physical Education, Henan University of Science and Technology, Luoyang, China; Ahvaz Jundishapur University: Ahvaz Jondishapour University of Medical Sciences, IRAN, ISLAMIC REPUBLIC OF

## Abstract

The study aims to investigate the impact of physical education teachers’ teaching beliefs and attributions of teaching success and failure on their effective teaching behaviors by examining typical quantitative relationships across three dimensions. Five cities in Henan Province were selected using a random sampling method. Within each city, two survey districts were randomly selected, and from each district six to eight primary and secondary schools were randomly chosen, culminating in a total of 64 surveyed primary and secondary schools. A total of 204 physical education teachers from primary and secondary schools were chosen to participate in this study. This research uses questionnaire surveys, semi-structured interviews, *t*- and *F*-tests, and canonical correlation analysis to analyse collected data. Results: Teaching experience emerged as a primary factor in influencing teaching beliefs, attributions of teaching success and failure, and effective teaching behaviors (*p <* 0.05). Teaching beliefs explained 53.2% of the variance in effective teaching behaviors. Attribution of teaching success accounted for 50.9% of this variance in effective teaching behaviors. While attributions of teaching failure explained 49.1% of this variance in effective teaching behaviors. Conclusion: The study recommends setting up a “mentorship system” for physical education teachers to acquire positive teaching experience. This system would enable senior teachers to mentor those with less teaching experience, which in turn motivates senior teachers to be more enthusiastic about teaching. Teachers should have the teaching belief of cultivating students’ success and the ability to communicate with students. Teachers should focus on internal attribution, which is more conducive to teaching success and more effective classroom outcomes. The study concludes that school administrative departments should focus on how teachers attribute their success and failure, and adopt strategies to improve the professional literacy of physical education teachers through promoting teaching reflection, providing regular training, and encouraging collaborative learning among educators.

## 1. Introduction

### 1.1. Research background

In physical education, the effectiveness of students’ acquisition of sports skills is closely linked to teachers’ teaching behaviors [1]. Physical education teachers strive to create an engaging teaching environment and develop proper plans to spark students’ interest in learning sports skills. To achieve these goals, teachers should employ a variety of effective teaching methods, strategies, and media to guide students in mastering these skills. This is the essential goal of physical education classes. An excellent physical education class benefits physical education teachers by allowing them to fully express their educational philosophies and fulfill their teaching potential. Moreover, it can also create a positive classroom atmosphere, enhance relationships between teachers and students, and continuously improve the effectiveness of students’ acquisition of sports skills. As a result, these efforts contribute to the successful accomplishment of physical education teaching goals. Given these dynamics, research on teaching conditions is a vital topic for physical education teachers, which not only contributes to their professional growth but also helps optimize educational functions.

In recent years, there has been a growing trend in examining the teaching process of physical education teachers from the perspective of cognitive psychology, with a particular focus on their psychological course. From the cognitive psychology perspective, physical education teachers aim to enhance teaching effectiveness to improve teaching quality. Based on teaching experience and theory, they update teaching methods, emphasize the design of teaching questions, reconstruct teaching content, clearly understand students, and dynamically evaluate their teaching behaviors. This leads to speculative teaching beliefs and attributions of teaching success or failure, which in turn promote a spiral improvement in their effective teaching behaviors. From this perspective, the transformation of curricula into actual teaching behaviors is viewed as an intermediary process that involves the teacher’s cognitive activity. In this process, teachers’ implicit theories and beliefs hold significant core values. To be specific, teaching beliefs and attributions of teaching success or failure are important implicit theories and beliefs that not only affect teachers’ thinking and decision-making but also influence their teaching behaviors [2]. When discussing the thought and behavior patterns of physical education teachers, some scholars have pointed out that these patterns include teachers’ attributions for student performance and their implicit theories of teaching and learning. These implicit theories and beliefs shape how teachers perceive and engage with the teaching process [3]. Furthermore, the intrinsic psychological courses involved in physical education teaching includes a range of teaching judgments and decision-making activities. Moreover, physical education teachers’ teaching beliefs and attributions are critical to their judgment and decision-making. Some scholars believe that physical education teachers’ teaching beliefs have a significant impact on students’ achievements in physical education [4]. Studies have shown that there is a close correlation between teaching beliefs and teaching efficacy, as well as students’ academic achievements. Teachers who possess positive teaching efficacy beliefs tend to exhibit superior teaching performance and achieve better outcomes compared to their counterparts with general efficacy beliefs [5]. Positive subject-specific teaching beliefs correlate with greater confidence in teaching performance. These beliefs influence lesson preparation, classroom management, and methods of imparting knowledge [6]. Research by Yang [7] has shown that providing students with cues, simplifying questions, and allowing them to modify certain parts of difficult tasks as supportive feedback can significantly boost their achievements in physical education.

The beliefs and attributions of teachers play a crucial role in the intrinsic psychological course of their teaching. Teachers’ views on students, curricula, textbooks, teaching methods, and their attributions of teaching success and failure, not only influence their judgments and decisions in educational activities but also affect their teaching behaviors in classroom interactions and reflective practices. Consequently, all these can even indirectly impact students’ learning achievements [1]. Some scholars assert that attributions of teaching success and failure are significant variables in predicting teachers’ self-efficacy in teaching. Teachers’ attributions are often shaped by their previous teaching experience. Successful teaching experiences may lead to internal and positive attributions, whereas past unsuccessful teaching experiences may result in external, negative attributions [8]. Therefore, different attribution modes can influence teachers’ teaching attitudes and approaches. For instance, attributing teaching success to personal teaching and failures to external factors such as students’ disengagement are significant variables in predicting teaching self-efficacy. Additionally, many scholars hold that cultural capital has a significant impact on physical education teachers’ teaching abilities and personal behaviors [9,10]. All indicate the common and specific characteristics in the teaching behaviors of physical education teachers. Moreover, studies on Wushu (Chinese martial arts) instructors reveal that their teaching processes involve various judgments and decisions closely linked to their teaching beliefs and attributions [11]. Similarly, the beliefs and attributions of physical education teachers are critical elements that guide their judgments and decisions in teaching situations [12].

Some scholars believe that the intensity of physical education classes should be progressive to ensure that the physical activities are challenging for most students, thereby boosting their engagement and motivation. Meanwhile, teachers should also guide students to seek meaningful and suitable ways of engaging in physical activities to maintain their involvement in PE classes [13]. Erlman [14] introduced the concept of “Informed Learner Pedagogy in Physical Education,” which is deemed as an effective for successful teaching. This pedagogy is characterized by four features: (1) Be familiar with the class content and educational priorities; (2) Guiding students to explore various topics within sports activities; (3) Assisting students in developing and executing plans; (4) Encouraging students to assess their outcomes. The teaching objective here is to empower students, helping them become “informed learners” of physical activities. Participation in planned teaching skills training not only helps physical education teachers improve their teaching effectiveness but also serves as a foundation for enhancing their teaching skills [15]. Excellent PE teachers excel by exploring teaching experiences from physical education, extracurricular sports training and competitions, and sports research. They continually refine their skills and expertise, progressing towards advanced levels of teaching proficiency. This development is marked by becoming “outstanding in PE teaching” and ultimately evolving into experts in the field. Additionally, environmental factors can influence teachers’ teaching behaviors [16]. For example, during the COVID-19 pandemic, PE teachers’ instructional practices were affected by the school environment and their own levels of anxiety [17].

A conclusion can be drawn from the above analysis that it is evident that teachers’ beliefs and attribution of success and failure play a significant role in the intrinsic psychological course of physical education. However, existing literature primarily focuses on qualitative research, with limited quantitative studies [18,19]. Canonical correlation analysis, with its ability to combine the strengths of correlation and regression for enhanced explanatory power, presents a promising method for examining these relationships. Therefore, future research should focus on exploring physical education teachers’ teaching beliefs and their attributions of teaching success and failure by employing canonical correlation analysis. clarify the extent of the influence of two variables on teaching beliefs, and the primary and secondary relationships between factors. Such research holds the potential to generate findings that can enhance the professional literacy of physical education teachers.

### 1.2. Research objectives

(1) To analyze the influence of demographic characteristics on teaching beliefs, attributions for teaching success or failure, and effective teaching behaviors of physical education teachers.
(2) To conduct a canonical correlation analysis between physical education teachers’ teaching beliefs and effective teaching behaviors.
(3) To conduct a canonical correlation analysis between the attribution of teaching success and effective teaching behaviors among physical education teachers.
(4) To conduct a canonical correlation analysis between the attribution of teaching failure and effective teaching behaviors among physical education teachers.

## 2. Methods

### 2.1. Demographic characteristics

The research targets physical education teachers in primary and secondary schools in Henan Province. Despite limited human and financial resources, a large-scale survey has been conducted to gain a comprehensive understanding of the teaching conditions for physical education teachers. The research period is from October 16th to December 26th, 2023. The survey is designed to include a diverse range of schools at different educational levels: primary schools, junior high schools, and high schools, employing scientific sampling methods. The research plan covers five cities, each with two urban districts, resulting in a total of ten urban districts. In each urban district, the survey targets three primary schools, two junior high schools, and one high school, summing up to 64 schools. The actual sampling would be adjusted according to the policies of the schools being surveyed. For the survey of teachers, the sampling is stratified by the teachers’ years of teaching experience, with a plan to sample 300 teachers. Specifically, the plan samples four teachers from each primary school, six from each junior high school, and five from each high school. However, the actual sampling would be adjusted according to the number of physical teachers available at each school.

The research employs a mixed-methods approach, combining both quantitative and qualitative research methods. The quantitative research primarily focuses on gathering survey data, which includes questionnaire surveys, descriptive analysis, independent samples t-tests, one-way ANOVA, LSD-tests, and canonical correlation analysis to derive meaningful research results. Using the random sampling method, we selected Luoyang, Zhengzhou, Xinxiang, Shangqiu, and Jiaozuo city as the research cities from Henan Province and then randomly selected 2 research areas from each city. Following the research plan, we randomly selected 6 primary and secondary schools from each of the 8 research areas and randomly selected 8 primary and secondary schools from each of the remaining 2 research areas (Table 1). A total of 64 schools (primary and secondary schools: 32 primary schools, 22 junior high schools and 10 high schools) were selected. The above research procedures were mainly based on online data, with validation from the local education department where necessary. Detailed information of the research was gathered as follows: Researchers established profiles for physical education teachers through consultation with personnel from school physical education departments. Subsequently, using purposive sampling based on years of experience in physical education teaching, we selected 300 physical education teachers as research subjects. A questionnaire survey including 300 physical education teachers was conducted through on-site distribution and collection of questionnaires within 60 minutes(Anonymous questionnaire). The questionnaire had to be completed following the order of the questions presented. A total number of 300 questionnaires were distributed, with 262 questionnaires returned. After removing incomplete responses, we retained 204 valid questionnaires for analysis.

**Table 1:** Physical education teachers’ demographic characteristics.

City	Region	Number of primary schools (Number of teachers)	Number of junior high schools (Number of teachers)	High school number of (Number of teachers)	∑	The effective number of people
Luoyang City	Jianxi District	4(12)	3(18)	1(3)	33	20
	Luolong District	4(12)	3(18)	1(5)	35	19
Zhengzhou City	Jinshui District	3(12)	2(12)	1(5)	29	23
	Huiji District	3(12)	2(12)	1(5)	29	25
Xinxiang City	Weibin District	3(12)	2(12)	1(5)	29	24
	Hongqi District	3(12)	2(12)	1(5)	29	22
Shangqiu City	Liangyuan District	3(12)	2(12)	1(5)	29	17
	Suiyang District	3(12)	2(12)	1(5)	29	18
Jiaozuo City	Jiefang District	3(12)	2(12)	1(5)	29	17
	Zhongshan District	3(12)	2(12)	1(5)	29	19

Qualitative research method mainly involves collecting empirical data from physical education teachers through semi-structured interviews in a planned manner (Adopt a one-on-one conversation approach with no third party present), with a focus on describing the behaviors, situational problems, and significance related to physical education curricula and its contextualization in daily life. From a total of 64 schools and 204 physical education teachers, 39 PE teachers were randomly selected for interviews based on school type. Specifically, the process involved randomly selecting one-half schools from each primary, middle, and high school levels, then randomly choosing one, one, and two teachers respectively from each level. Additionally, from one middle school known for its distinctive physical education program, three teachers were randomly selected. The interview explored various aspects of the teachers’ professional perspectives, including their teaching beliefs, attributions regarding teaching successes and failures, effective teaching behaviors, and experiences in physical education, and factors that influence and promote teaching. Physical education teachers were encouraged to share insights selectively based on their personal experiences.The final sample included 204 physical education teachers from primary and secondary schools in Henan Province, aged between 23 and 46 (consisting of 92 males and 112 females. Assigned values in order 1,2). Their teaching experience in physical education varied as follows: 48 teachers had ≤ 5 years of experience, 70 teachers had 6–10 years of experience, 70 teachers had 11–15 years of experience, and 16 teachers had 16–20 years of experience, assigned values in order 1, 2, 3, 4. In terms of education background, 27 teachers had an Associate Degree or below, 107 teachers had a Bachelor’s degree, and 70 teachers had a Master’s degree or higher, assigned values in order 1, 2, 3.

### 2.2. Research tools

Referring to the study conducted by Li and Zhang [20], the survey questionnaire has been revised to align with the research objective and is now titled “*Investigation Questionnaire on the Influence of Physical Education Teachers’ Teaching Beliefs, Attributions of Teaching Success and Failure on Their Effective Teaching Behavior*” based on the research objective. This questionnaire comprises four sub-questionnaires: “*Investigation Questionnaire on Teaching Beliefs in Physical Education*,” *“Investigation Questionnaire on Attributions of Teaching Success in Physical Education*,” *“Investigation Questionnaire on Attributions of Teaching Failure in Physical Education,*” and “*Investigation Questionnaire on Effective Teaching Behavior in Physical Education (abbreviated as Effective Teaching Behavior).*” To ensure high content validity, the sub-questionnaires were meticulously revised three times by a panel of eight physical education teachers (The 8 physical education teachers are renowned in physical education and possess rich teaching experience). Following these revisions, a pre-test was conducted with 86 teachers to assess the reliability of the survey instruments. The reliability is assessed using Cronbach’s α value, a statistical measure that denotes internal consistency. The resulting of Cronbach’s α values for the four sub-questionnaires ranges from 0.836 to 0.875, indicating a high degree of reliability. These values demonstrates that the questionnaires consistently measure the underlying constructs they are intended to assess, proved to be reliable tools for this research study.

The “*Investigation Questionnaire on Teaching Beliefs in Physical Education*” includes four dimensions: competence flexibility, student diversity, efficacy, and trust, each with four item factors. The Cronbach α value for the questionnaire is 0.824.

Similarly, both “Investigation Questionnaire on Attributions of Teaching Success in Physical Education,” and “Investigation Questionnaire on Attributions of Teaching Failure in Physical Education,” consist of four dimensions: teachers’ teaching, physical and mental states, assistance from others, and student factors, each with four item factors. The Cronbach α values for questionnaire are 0.833 and 0.855, respectively.

The “*Effective Teaching Behavior*” consists of three dimensions: teaching planning, teaching interaction, and teaching atmosphere, each with five item factors. The Cronbach α value for the questionnaire is 0.881.

In terms of format and scoring, the questionnaire employs a five-point Likert scale, ranging from “completely consistent” (extremely important) to “completely inconsistent” (extremely unimportant). Scores ranges from 5 to 1, with higher scores indicating greater agreement. Specifically, a higher score indicates a stronger alignment with teaching beliefs in physical education, a greater tendency to attribute teaching success and failure to these beliefs, and a higher degree of embodying effective physical education behaviors. In contrast, lower scores suggests poor alignment with these teaching beliefs, a weaker tendency to attribute teaching success and failure to them, and less possibility to embody effective teaching behaviors.

Data were collected from 204 physical education teachers from primary and secondary schools in Henan province. These questionnaires are analyzed by using SPSS20 statistical software. The factor skewness ranges from 1.409 to 0.094, and factor kurtosis ranges from 0.002 to 2.603. This analysis confirms that the data follow a normal distribution, consistent with statistical norms.

### 2.3. Research methods

The framework of this study is established through a literature review, with survey data and supporting evidence obtained through questionnaire surveys and interviews. The collected data are analyzed using SPSS20 software, employing various statistical methods including descriptive analysis, independent samples *t*-tests, one-way *ANOVA*, *LSD*-tests, and canonical correlation analysis to derive meaningful research results. Given that the variables in the study consist of two sets of multivariate data rather than single-variable data, it is advisable to employ canonical correlation analysis for a comprehensive depiction of the relationship between these two sets of variables. Canonical correlation analysis allows for the extraction of information from both variable sets using multiple sets of canonical variables. This analytical method ensures that the derived correlations effectively represent the original relationships between the two data sets. it is advisable to employ canonical correlation analysis for a comprehensive depiction of the relationship between these two sets of variables. Canonical correlation analysis allows for the extraction of information from both variable sets using multiple sets of canonical variables. This analytical method ensures that the derived correlations effectively represent the original relationships between the two data sets. Within the context of canonical correlation analysis, when the signs (positive or negative) of the two groups of multivariate data align in the same direction, a same-direction sign argument can be employed.

### 2.4. Statistical analysis

The data are analyzed using SPSS version 20.0. All tests are performed with bilateral α-level of < 0.05 to indicate statistical significance.

### 2.5. Ethics approval and consent to participate

The research project does not include any animal experiments or human drug experiments. It is a public welfare project, and informed consent had been obtained before the research. After discussion by the ethics committee of the School of Physical Education, Henan University of Science and Technology, the project research was approved (Project No: 20231015). Before participating in the study, each participant’s written informed consent had been solicited.

## 3. Result

### 3.1. The influence of demographic characteristics on all dimensions of physical education teachers’ teaching beliefs, attributions of teaching success and failure, and effective teaching behaviors

Table 2 displays the results of t-tests and F-tests conducted for each factor. The analysis reveals that, at the student level, there is a statistically significant difference in teaching success among physical education teachers based on gender (*p* < 0.05). However, no significant differences have been found across various dimensions of teaching beliefs, attributions for teaching success and failure, and effective teaching behaviors when considering educational level (*p* > 0.05).

**Table 2:** Summary of the Impact of Demographic Characteristics on Physical Education Teachers’ Teaching Beliefs, Attributions of Teaching Success and Failure, and Effective Teaching Behaviors.

Factor	Factor	Gender		Education background		Teaching experience		
		*t*	*p*	*F*	*p*	*F*	*p*	*LSD-test*
Teaching beliefs	Competence flexibility	0.252	0.801	0.095	0.909	2.841	0.039*	4>1**;4>2*
	Efficacy	1.198	0.232	1.289	0.278	4.267	0.006**	4>1*;4>2**;3>2**
	Student diversity	-0.874	0.383	0.879	0.417	2.204	0.089	
	Trust	0.505	0.614	0.091	0.913	3.407	0.019*	4>1*;3>2*;4>2**
Teaching success	Student factors	2.214	0.028*	0.862	0.424	4.142	0.007**	4>1*;3>2**;4>2*;3>1*
	Teachers teaching factors	1.617	0.108	0.101	0.904	6.056	0.001**	4>1**;3>2**;4>2*;3>1*
	Physical and mental states	1.369	0.172	0.982	0.376	3.882	0.010*	4>2**;4>1;3>2*
	Assistance from others	-0.872	0.384	1.098	0.335	3.195	0.025*	3>1*;4>1*;4>2*;3>2*
Teaching failure	Student factors	-0.771	0.422	0.056	0.946	1.786	0.151	
	Teacher Teaching Factors	1.860	0.064	1.316	0.271	3.424	0.018*	4>2*;3>2**
	Physical and mental states	0.331	0.741	0.781	0.459	1.952	0.122	
	Assistance from others	0.421	0.674	0.190	0.827	1.925	0.127	
Efficient Teaching Behaviors	Teaching plan	1.809	0.072	1.002	0.369	2.898	0.036*	3>2*;4>2*
	Teaching atmosphere	1.164	0.246	0.520	0.595	1.812	0.146	
	Teaching interaction	0.619	0.537	0.114	0.892	3.274	0.022*	3>1*;4>1*;3>2*

Note: *p<0.05, **p<0.01, ***p<0.001. Symbolic expression of teaching experience:≤ 5 years of experience(Symbol 1), 6–10 years of experience(Symbol 2), 11–15 years of experience(Symbol 3), 16–20 years of experience(Symbol 4).

Conversely, years of teaching experience are associated with significant differences in several dimensions of teaching success (*p* < 0.05, *p* < 0.01), effective teaching behaviors only shows statistically no significant difference in the dimension of teaching atmosphere (*p*>0.05). Competence flexibility, efficacy, and trust in teaching beliefs also vary significantly with teaching experience (*p* < 0.05, *p* < 0.01). Teaching failure only shows statistically significant difference in the dimension of teaching factors (*p* < 0.05). Further examination by LSD shows that teachers with longer teaching experience are more influential than those with shorter teaching experience.

These findings indicate that physical education teachers’ gender and education background are not the primary factors in influencing their teaching beliefs, attributions of success or failure, and effective teaching behaviors. Rather, teaching experience plays a critical role in influencing these factors.

### 3.2. Canonical correlation analysis of physical education teachers’ teaching beliefs and effective teaching behaviors

A comprehensive canonical correlation analysis has been conducted on the four dimensions of physical education teaching beliefs and the three dimensions of effective physical education teaching behaviors, as shown in Table 3. Significant testing of canonical functions reveals three sets of canonical factors, with only one set demonstrating statistical significance (Wilk’s λ = 0.278, *p* < 0.001).

**Table 3:** Summary of Canonical Correlation Tests.

Canonical correlation	Wilk’s	Chi-SQ	DF	Sig.
1	0.278	393.255	12.000	0.000***
2	0.971	9.034	6.000	0.172
3	0.998	0.670	2.000	0.715

Note: *p<0.05, **p<0.01, ***p<0.001.

According to Table 4, the correlation coefficient for the first set of canonical functions (χ1, η1) is ρ=0.845, indicating that the first set of canonical factor of teaching beliefs χ1 can explain 71.4% of the total variance in the first canonical factor of effective teaching behaviors η1.

**Table 4:** Summary of canonical correlation analysis between various dimensions of teaching beliefs and effective teaching behaviors.

X variable	Typical factors	Y variable	Typical factors
(Teaching beliefs)	χ1	(Effective teaching behaviors)	η1
Competence flexibility	-0.963	Teaching plan	-0.768
Efficacy	-0.751	Teaching atmosphere	-0.850
Students Diversity	-0.704	Teaching interaction	-0.961
Trust	-0.580		
Overlap coefficient	0.415	Overlap coefficient	0.532
Extracting ANOVA percentage	0.581	Extracting ANOVA percentage	0.745
		The canonical correlation coefficient(*ρ*)	0.845***
		The typical root value (*ρ*^2^)	0.714

Note: *p<0.05, **p<0.01, ***p<0.001.

When examining the variables individually, the canonical factor χ1 of teaching beliefs accounts for 58.1% of the total variance across all dimensions of teaching beliefs. Similarly, the canonical factor η1 explains 74.5% of the variance across all dimensions of effective teaching behaviors. These results indicate that the canonical factor χ1 effectively represents four observed variables: competence flexibility, efficacy, student diversity, and trust. On the other hand, η1 fully represents three observed variables: teaching interaction, teaching atmosphere, and teaching planning.

The four dimensions of teaching beliefs primarily influence the three dimensions of effective teaching behaviors through the canonical factors (χ1,η1), explaining 53.2% of the total variance in effective teaching behaviors. Conversely, the three dimensions of effective teaching behaviors primarily influence the four dimensions of teaching beliefs through the canonical factors (η1,χ1), explaining 41.5% of the total variance in teaching beliefs. Furthermore, the importance of each dimension of teaching beliefs in the canonical factors ranks in the following order: competence flexibility (-0.963), efficacy (-0.751), student diversity (-0.704), and trust (-0.580). Similarly, the importance of each dimension of effective teaching behaviors in the canonical factors ranks in the following order: teaching interaction (-0.961), teaching atmosphere (-0.850), and teaching planning (-0.768).

Based on the factor loadings, it can be inferred that there is a positive correlation between teaching beliefs and effective teaching behaviors. That is, the higher the teaching beliefs physical education teachers hold, the better their effective teaching behaviors demonstrate, with the most significant impact observed between competence flexibility and teaching interaction.

### 3.3. Canonical correlation analysis between the various dimensions of attributions of teaching success and effective teaching behaviors in physical education

A comprehensive canonical correlation analysis has been conducted to examine the relationship between the four dimensions of attributions of teaching success in physical education and the three dimensions of effective teaching behaviors (as shown in Table 5). From this analysis, three sets of canonical factors are identified. However, the significance test for the canonical functions reveals that only the first set of canonical factors demonstrates statistical significance (Wilk’s λ = 0.329, *p* < 0.001).

**Table 5:** Summary of the canonical correlation significance test.

Canonical correlation	Wilk’s	Chi-SQ	DF	Sig.
1	0.329	341.208	12.000	0.000***
2	0.985	4.601	6.000	0.596
3	0.999	0.435	2.000	0.805

Note: *p<0.05, **p<0.01, ***p<0.001.

According to Table 6, the correlation coefficient for the first set of canonical functions (χ1, η1) is ρ= 0.816, indicating that the first set of canonical factors χ1 of the attributions of teaching success, can explain 66.6% of the total variance in the first set of canonical factors η1 of effective physical education behaviors.

**Table 6:** Summary of the canonical correlation analysis between various dimensions of attributions of teaching success and effective teaching behaviors.

X variable	Canonical factors	Y variable	Canonical factors
Attributions to Teaching Success	χ1	Effective teaching behaviors	η1
Student factors	-0.909	Teaching plan	-0.808
Teacher teaching factors	-0.921	Teaching atmosphere	-0.871
Physical and mental states	-0.903	Teaching interaction	-0.939
Assistance from others	-0.874		
Overlap coefficient	0.542	Overlap coefficient	0.509
Extracting ANOVA percentage	0.814	Extracting ANOVA percentage	0.765
		The canonical correlation coefficient(*ρ*)	0.816***
		The typical root value (*ρ*^2^)	0.666

Note: *p<0.05, **p<0.01, ***p<0.001.

In explaining the variables, the canonical factor χ1 of attributions of teaching success accounts for 81.4% of the total variance across all dimensions of teaching success attributions. Similarly, the canonical factor η1 of effective teaching behaviors accounts for 76.5% of the total variance across all dimensions of effective teaching behaviors. This result indicates that canonical factor χ1 can represent the four observed variables: student factors, teacher’s teaching factors, physical and mental states, and external assistance from others. Correspondingly, the canonical factor η1 can fully represent three observed variables: teaching plan, teaching atmosphere, and teaching interaction.

The four dimensions of attributions of teaching success primarily influence the three dimensions of effective teaching behaviors through canonical factors (χ1, η1), and explain that effective teaching behaviors account for 50.9% of the total variance. Similarly, the three dimensions of effective teaching behaviors primarily impact the four dimensions of attributions of teaching success through canonical factors (η1, χ1), and explain that attributions of teaching success account for 54.2% of the total variance. Furthermore, the importance of each dimension of attributions of teaching success in the canonical factors ranks in the following order: teacher teaching factors (-0.921), student factors (-0.909), physical and mental states (-0.903), and assistance from others (-0.874). Similarly, the importance of each dimension of effective teaching behaviors in the canonical factors ranks in the following order: teaching interaction (-0.939), teaching atmosphere (-0.871), and teaching plan (-0.808).

Based on the factor loadings, it can be concluded that there is a positive correlation between attributions of successful teaching and effective teaching behaviors. In other words, the stronger the attributions of teaching success held by physical education teachers, the better their effective teaching behaviors. with the most significant influence observed between the teaching factors and teaching interactions.

### 3.4. Canonical correlation analysis of various dimensions of attributions of teaching failure and effective teaching behaviors in physical education

A comprehensive canonical correlation analysis has been conducted on four dimensions of attributions of teaching failure and three dimensions of effective education behaviors in physical education (as presented in Table 7). The analysis identifies three sets of canonical factors through the significance test of canonical functions, but only two groups exhibit statistical significance. (Wilk’s λ= 0.332, *p* < 0.001; Wilk’s λ= 0.937, *p* < 0.05).

**Table 7:** Summary of canonical correlation significance test.

canonical correlation	Wilk’s	Chi-SQ	DF	Sig.
1	0.332	219.317	12.000	0.000***
2	0.937	12.930	6.000	0.044*
3	0.982	3.528	2.000	0.171

Note: *p<0.05, **p<0.01, ***p<0.001.

Based on Table 8, the correlation coefficients between the two sets of canonical functions (χ1, η1) and (χ2, η2) are ρ=0.803 and *ρ*=0.215, respectively. This indicates that the first set of canonical factors χ1 of attributions of teaching failure can explain that the first set of canonical factors η1 of effective teaching behaviors account for 64.5% of the total variance. In contrast, the second set of canonical factors χ2 of attributions of teaching failure can explain that the second set of canonical factors η2 of effective teaching behaviors account for 4.6% of the total variance. However, the analysis primarily focuses on the first set of canonical correlations.

**Table 8:** Summary of the canonical correlation analysis between various dimensions of attributions of teaching failure and effective teaching behaviors.

X variable	Canonical factors	Canonical factors	Y variable	Canonical factors	Canonical factors
(Attributions of teaching failure)	χ1	χ2	(Effective teaching behaviors)	η1	η2
Student factors	-0.877	0.179	Teaching interaction	-0.789	-0.430
Teacher teaching factors	-0.742	0.154	Teaching atmosphere	-0.874	-0.342
Physical and mental states	-0.820	-0.216	Teaching plan	-0.933	0.334
Assistance from others	-0.785	-0.609			
Overlap coefficient	0.421	0.005	Overlap coefficient	0.485	0.006
Extracting ANOVA percentage	0.652	0.119	Extracting ANOVA percentage	0.752	0.138
			The canonical correlation coefficient(*ρ*)	0.803***	0.215*
			The typical root value (*ρ*^2^)	0.645	0.046

Note: *p<0.05, **p<0.01, ***p<0.001.

In terms of explaining variables, the first set of canonical factors (χ1) of attributions of teaching failure accounts for 65.2% of the total variance in attributions. The second set (χ2) accounts for 11.9% of the total variance in attributions. Together, these factors (χ1, χ2) explain 77.1% of the variance across four observed variables: student factors, physical and mental states, external assistance, and teacher’s teaching factors. For all dimensions of effective teaching behaviors, the first set of canonical factors (η1) explains 75.2% of the total variance, while the second factor (η2) explains 13.8% of the variance. Combined, these canonical factors (η1, η2) account for 89% of the total variance across three observed variables: teaching interaction, teaching atmosphere, and teaching plan. This result indicates that the two sets of canonical factors derived from teaching failure effectively represent the observed variables (student factors, physical and mental states, external assistance, and teachers’ teaching factors), and η1 represents the teaching plan, teaching atmosphere, and teaching interaction.

The four dimensions of attributions of teaching failure primarily influence the three dimensions of effective teaching behaviors through canonical factors (χ1,η1), explaining 48.5% of the total variance in effective teaching behaviors. Similarly, the second set of canonical factors (χ2,η2) of attributions of teaching failure explains only 0.6% of the variance in effective teaching behaviors. Together, these two sets of canonical factors (χ1,η1, and χ2,η2) explain 49.1% of the total variance in effective teaching behaviors.

Conversely, the three dimensions of effective teaching behaviors primarily influence the four dimensions of attributions of teaching failure through canonical factors (η1,χ1), explaining 42.1% of the total variance in attributions. The second set of canonical factors (η2,χ2) accounts for only 0.5% of the variance. Together, these two sets of factors explain 42.6% of the total variance in attributions of teaching failure.

In the first set of factor analysis, the importance of each dimension of attributions of teaching failure and effective teaching behaviors ranks in the following order: student factors (-0.877), physical and mental states (-0.820), assistance from others (-0.785), and teacher teaching factors (-0.742). The importance of each dimension of effective teaching behaviors ranks in the following order: teaching planning (-0.933), teaching atmosphere (-0.874), and teaching interaction (-0.789). Based on the factor loadings, it can be inferred that the higher the attributions of teaching failure held by physical education teachers, the better their effective teaching behaviors, with the most significant influence observed between student factors and teaching plans.

In the analysis of the second set of canonical factors, there is a significant positive correlation between external assistance (-0.609) and teaching interactions (-0.430).

## 4. Discussion

### 4.1. The influence of demographic characteristics on all dimensions of physical education teachers’ teaching beliefs, attributions of teaching success and failure, and effective teaching behaviors

Teaching experience is a primary factor in influencing teaching beliefs, attributions of teaching success, and effective teaching behaviors. Over time, teachers generally progress through various stages, including novice adaptation, growth, flourishing, stagnation, and development. Their teaching efficacy evolves with the accumulation of experience [21]. Some scholars claim that, over time, teachers improve their professional competence and adopt more scientific attributions for teaching success and failure [22]. Most research shows a statistical significance in learning beliefs and teaching efficacy among teachers with different years of teaching experience, with more experienced teachers often exhibiting stronger teaching beliefs and higher teaching efficacy [3,23]. However, this study found that most factors attributed to teaching failure did not show statistical significance in teaching experience. This discrepancy may be due to differences in the study subjects and the teaching culture of the surveyed region. According to interviews, physical education teachers are tasked with cultivating students’ moral character, creating practical teaching activities, improving teaching methods through feedback from educational research meetings, and enhancing their professional competence through various learning opportunities. Therefore, there was no statistical significance in all dimensions of teaching beliefs and effective teaching behaviors, except for student-related factors influencing attributions of teaching success and failure.

Upon reviewing the literature, it has been found that research shows gender-based differences in attributions for teaching failures. Female teachers tend to attribute their failures to poor competence, while male teachers attribute their failures to insufficient effort and dedication. These differences lead to variations in their teaching efficacy and teaching beliefs [24]. From a professional perspective, higher levels of education are expected to correlate with stronger professional competence, which in turn affects teaching efficacy and attributions of teaching success and failure [25,26]. However, research findings can vary due to differences in the demographic characteristics, regions, and sample sizes of the subjects involved.

This study suggests that to improve the effectiveness of physical education teaching, teachers with longer teaching experience should systematically summarize their teaching experience and pass them on to younger teachers to promote effective teaching behavior. The physical education team should establish a mentoring system for teaching guidance to help young teachers enhance their teaching beliefs and improve the factors that promote teaching success.

### 4.2. Representative cases

#### 4.2.1. Case study of A2.

A2 is a physical education teacher at a junior high school with 11 years of teaching experience. Early in his career, he concentrated on honing the athletic skills of a few exceptional students, aiming for their participation in athletic competitions. However, this approach received a negative response from most students in his class. Over time, he developed unique insights into physical education. He now believes the class content should be accessible to most students, providing them with opportunities for reflection, practice, and discussion. He encourages students to showcase their athletic skills at appropriate times while differentiating between advanced and less advanced students. His goal is to challenge the more skilled students and foster a sense of progress among those less advanced.

#### 4.2.2. Case study of A6.

A6 is an outstanding physical education teacher in primary schools. She posits that a teacher’s compassion, dedication, and sense of responsibility are crucial for successful teaching outcomes. In her view, teaching experience acts as a catalyst, enhancing the effectiveness of classroom instruction.

#### 4.2.3. Case study of A25.

A25, a high school physical education teacher, acknowledges the significant impact of physical education on students. He emphasizes the importance of integrating rich, real-life content into his teaching to help students better understand and engage with the subject matter. However, he finds it challenging to consistently identify suitable, everyday-life-relevant content due to his limited experience.

#### 4.2.4. Case study of A31.

A31 is an accomplished physical education teacher with 8 years of experience, teaching both elementary and junior high school students. She recognizes the diverse abilities among students in terms of intelligence, cognitive capacity, comprehension, and acceptance. As such, she sets varied expectations for her students. For example, she requires more capable students to meet higher standards for certain motor skills, whereas less capable students are only expected to demonstrate basic competency. Acknowledging that student progress varies, she tailors her guidance and expectations to each individual’s circumstances, aiming to ensure that every student benefits from the classroom experience.

### 4.3. Canonical correlation analysis of various dimensions of teachers’ teaching beliefs and effective teaching behaviors in physical education

Research indicates that teachers who believe in the potential for student abilities to improve through education tend to prioritize individual differences in their teaching. They adopt varying standards and promote a physical education framework that positively impacts student outcomes. These teachers perform better in effective teaching behaviors such as teaching planning, interaction, and creating a positive teaching atmosphere.

The teaching beliefs of physical education teachers determine their teaching behavior. These beliefs not only affect their choices and decisions but also guide their selection of teaching strategies and behaviors. While internal beliefs are crucial, the external environment also plays a vital role in shaping teaching beliefs [27]. Some researchers suggest that the evolution of teaching beliefs and the rationality of teaching behavior follow a similar trend, and teaching beliefs will affect teachers’ teaching behavior to a certain extent [1]. Yan’s research results show that the relationship between physical education teachers’ teaching beliefs and their classroom teaching is bidirectional. Different beliefs will guide teachers to adopt various problem-solving methods [28]. The robustness and quality of teachers’ teaching beliefs can have a profound impact on their teaching decisions and teaching behavior, because these teachings directly influence how teachers define tasks, choose methods, employ strategies, analyze materials, and manage their classrooms.

In terms of efficacy, relevant research has found that the teaching efficacy beliefs of physical education teachers have a significant influence on their effective teaching behaviors and students’ academic achievements. Physical education teachers with high teaching efficacy beliefs tend to demonstrate more effective teaching behaviors. For instance, Wang and other scholars hold that teaching efficacy, as an intrinsic belief of teachers, is a crucial factor that influences their thought and behaviors [29]. Some researchers argue that teachers’ teaching beliefs play a strongly positive role in terms of execution, content, and structural modes. They contend that these beliefs are more influential than knowledge itself, making them the most critical factor in guiding teaching practices [30].

The teaching beliefs of physical education teachers in terms of students’ diversity and trust can influence their teaching behaviors. Research indicates that physical education teachers with strong teaching beliefs in student diversity and trust show more tendency to prioritize individual differences and trust their students. They are committed to designing practical physical education materials, teaching plans, and teaching activities that drive students to learn proactively. As a result, they are more excellent at teaching planning, teaching interaction, and teaching atmosphere.

In terms of competence flexibility, it can influence the effective teaching behaviors of physical education teachers. Teachers who strongly believe in their ability to cultivate student potential tend to adopt an optimistic attitude toward physical education. They are more willing to engage in various teaching behaviors that enhance the effectiveness of their instruction.

To support this, systematic in-service training and symposia for physical education teachers are essential. These should provide a comprehensive support system to enhance professional competence, address teaching challenges, and boost teachers’ confidence in the effectiveness of physical education. Regular introspection and continuous improvement of teaching beliefs can help teachers develop a positive and proactive outlook, leading to more effective teaching behaviors and improved educational outcomes. Well-planned in-service training and symposia are crucial for professional development, allowing physical education teachers to enhance their skills, tackle challenges, and reinforce their belief in the effectiveness of their teaching practices. This, in turn, promotes excellent performance and improves the overall efficacy of physical education programs.

Teachers’ teaching beliefs should be based on students’ personality traits and growth patterns, with students as the center, to achieve better effectiveness of “teaching” and “learning”.

However, some literature shows discrepancies between physical education teachers’ teaching beliefs and their actual teaching behaviors. While teachers often claim to prioritize students’ autonomy and development, they tend to use direct teaching methods in practice. Research has shown that teachers rarely adjust their teaching based on students’ reactions, and they primarily rely on one-way indoctrination rather than two-way interaction. Teachers may fail to keep consistent with their teaching beliefs due to changes in teaching behavior over time [31]. In addition, physical education teachers’ teaching beliefs are influenced by their teaching experience, educational level, gender, workload, student characteristics, peer interaction, and school culture [32].

### 4.4. Representative cases

#### 4.4.1. Case study of A3.

A3 is a martial arts teacher in primary school who teaches Taijiquan. Initially, Teacher A3 faced challenges with students’ disinterest in Taijiquan due to its slow movements. To address this, A3 incorporated offensive and defensive skills into the lessons, making the class more engaging by allowing students to experience the combative aspects of Taijiquan. This adjustment resulted in a livelier classroom environment and significantly improved teaching effectiveness. A3 believes that innovative teaching methods are crucial for enhancing students’ learning outcomes.

#### 4.4.2. Case study of A9.

Teacher A9 believes that “children in the lower grades of primary school are lively and active, and their attention is easily distracted, so it is necessary to choose teaching methods that can capture students’ interest and allow them to learn physical education knowledge through games. Moreover, children in the primary school stage should be encouraged more. A9 provides timely verbal praise to encourage students, boost their self-confidence, and increase class participation. This approach fosters a positive and engaging learning environment.”

#### 4.4.3. Case study of A12 and A22.

Teachers A12 and A22 are aerobics coaches at junior high schools. In the 2020–2021 school year, Teachers A12 and A22 stuck to a rigid teaching plan for aerobics that focused on skill acquisition without emphasizing precision or rhythm, leading to low skill assessment scores. In 2022, they introduced a new teaching element where students watched and analyzed aerobics competition videos every two classes. By explaining the distinctive characteristics and advantages of the athletes’ movements, they allowed students to experience and understand the nuances of the sport. This change led to a significant improvement in teaching effectiveness in 2022: students’ assessment scores soared, and a team of eight students even won bronze medals in a provincial competition.

### 4.5. Canonical correlation analysis of various dimensions of attributions of teaching success and effective teaching behaviors in physical education

Effective teaching behaviors in physical education are influenced by four key factors: teachers’ teaching, physical and mental states, assistance from others, and student factors. Among these, teachers’ teaching factors are the most influential. Teachers who attribute their teaching success to their abilities and efforts, their physical and mental states, their collaboration with colleagues, and the abilities and efforts of their students tend to exhibit effective teaching behaviors. These behaviors are evident in various aspects, including lesson planning, teacher-student interactions, and the overall classroom environment.

Some scholars believe that the teaching success of physical education can be attributed to individual teaching factors. The teaching behaviors of physical education teachers are the primary factors that affect their teaching effectiveness [33]. However, factors related to the students and societal norms are also critical to the success of physical education [34]. Moreover, from the perspective of attribution theory, individuals who credit their success to factors within their control are likely to experience pride and an increased sense of self-worth. This, in turn, motivates them to persist in their efforts. Therefore, teachers’ teaching attributes in physical education are often seen as the most influential factors in driving effective teaching behaviors. When teachers attribute their success to their efforts and abilities, it reflects an internally controlled viewpoint, which enhances their self-worth and encourages them to maintain good teaching practices. Conversely, some scholars point out that if teachers attribute learning success to students’ innate talent or external factors, they emphasize ability over effort. This can negatively impact their teaching self-efficacy. Teachers who make internal, stable, and controllable attributions for their success tend to have a higher sense of teaching self-efficacy [35]. Thus, physical education teachers need to focus on developing students’ abilities while maintaining a flexible approach to teaching.

Yi and colleagues, approaching from a student’s perspective, discovered that over 90% of students believe that an outstanding physical education teacher creates opportunities for them to think, practice motor skills, and express themselves. Additionally, 86% of students perceive successful physical education classes to be those where they learn sports skills amid an active classroom atmosphere [36]. The teaching behavior of a teacher is influenced by the classroom environment and the student’s knowledge and skills. However, experienced teachers effectively utilize teaching techniques to motivate students to learn proactively by analyzing the current physical education classroom situation [37]. Effective teaching behaviors in physical education can significantly enhance students’ enthusiasm for learning and reduce disruptive classroom behaviors [38]. Teachers who attribute their success to their emotional state actively manage their emotions during teaching activities and approach teaching with optimal mental states [39]. Teachers with high teaching efficacy tend to attribute their success to internal or controllable factors. Furthermore, the attribution of physical education teachers’ success or failure is influenced by a range of factors, including their teaching experience, educational level, gender, workload, student characteristics, interaction with colleagues, and school culture [40].

Furthermore, research indicates that the factors contributing to teaching success can be viewed from four perspectives, all of which are closely related to the teaching behaviors of physical education teachers, thereby influencing their effectiveness. A physical education teacher’s ability and efforts directly impact the quality of their teaching, while their physical and mental states indirectly influence their engagement in teaching. Additionally, in terms of assistance from others, teaching experience shared by experienced physical education teachers helps them improve. Therefore, the exchange of experiences among teachers is a critical factor in attributing success in physical education teaching. Moreover, active student cooperation helps teachers complete their teaching tasks, thus influencing effective teaching behaviors. In addition, students’ active cooperation can help teachers complete teaching tasks, which also affects effective teaching behaviors.

Therefore, teacher training institutions and educational administrative agencies must understand how physical education teachers attribute their teaching success. They should offer appropriate teaching guidance, assist teachers in addressing practical teaching challenges, and help establish correct views on the attributions of teaching success in physical education. Teachers should hold the belief that students can be molded and developed. They should be capable of communicating effectively with different students, and consistently managing positive emotions in classroom. These aspects are beneficial to teaching success and promote effective teaching behavior.

### 4.6. Representative cases

#### 4.6.1. Case study of A14.

Teacher A14, an elementary school football coach and an outstanding educator, mentors the football team which trains twice a week. He believes that a successful football classroom is contingent upon the rapport between the teacher and the students. Factors such as the teacher’s professional competence, the student’s level of concentration, and the exchange of experiences between teacher and student all play significant roles in this rapport. During the interview, Teacher A14 emphasized that he rejects introducing negative emotions into the classroom, as it is a space that holds emotional value and should not be compromised by negative sentiments.

#### 4.6.2. Case study of A21.

A21 is a high school physical education teacher and another exceptional educator. In each of his physical education classes, he engages students in learning ‘problems’ and then guides them to learn around the goal of solving these ‘problems.’ He believes that if educators can center their teaching around clear objectives, each teaching method and activity will have defined goals, and the practice of student skills will be goal-oriented. This approach ensures a strong sense of direction in teaching and learning. Naturally, educators should meticulously craft questions and challenges throughout the course, aiming to enlighten students’ minds and enhance their understanding of sports knowledge through thinking, practice, and responses.

#### 4.6.3. Case study of A28.

A28 is a physical education teacher at a Junior High School, she recognizes that the diversity and heterogeneity of students lead to distinct needs and developmental potential. Her lessons are structured to cater to students with lower physical fitness levels with “general knowledge” instruction, and those with higher fitness levels with “challenge-oriented” content. She provided an illustrative example of the classroom framework: when teaching the high jump, she first imparts fundamental knowledge to students, enabling them to master the technique through practice. Subsequently, she guides students towards standardized movements, culminating in class competitions among students of varying fitness levels. This approach ensures that each student experiences a sense of accomplishment within the physical education classroom.

#### 4.6.4. Case study of the physical education team at B junior high school.

The physical education team at B Junior High School (comprising three teachers) attributes successful classroom instruction to the group’s educational research and teaching activities. They convene twice a month to collectively discuss pedagogical issues and enhance classroom effectiveness. These meetings provide a platform for teachers to address problems in physical education and share effective teaching methods, thereby contributing to the professional development of physical education teachers and improving the quality of teaching.

### 4.7. Canonical correlation analysis of various dimensions of attributions of teaching failure and effective teaching behaviors in physical education

Effective teaching behaviors in physical education are influenced by four factors: teachers’ teaching, physical and mental states, assistance from others, and student factors. Among these, student factors are the most influential. According to data presented in Table 7, physical education teachers do not reduce their efforts to maintain effective teaching behaviors, even in the face of failures in physical education classes. In light of this finding, interviews with 98 respondents revealed that 98.98% of teachers believe that a single failure in physical education class should not weaken their future teaching efforts. Instead, physical education teachers will conscientiously introspect reasons for teaching failures and then further improve and make plans for future physical education curricula. The aim is not to weaken teaching behaviors but rather to effectively promote better teaching performance. This conclusion demonstrates the noble professional ethics of physical education teachers.

From the perspective of attribution theory, when individuals attribute their failure experience to themselves, it is likely to evoke feelings of self-blame, which can damage their self-esteem and subsequently lead to self-devaluation. Therefore, when discussing the impact of student factors on effective teaching behaviors, physical education teachers tend to attribute teaching failures to student factors to safeguard their self-esteem. As a result, the impact of student factors on effective teaching behavior is more pronounced. This tendency is linked to the psychological concept of teachers’ maintaining their self-esteem. However, this does not weaken the effective teaching behaviors of physical education teachers. Instead, it produces a positive teaching attitude, improves physical education teaching plans, and incorporates new teaching techniques to promote an organized teaching process and stimulate students’ intrinsic learning motivation. This argument has been approved through interviews.

Research reveals that the four dimensions of attributions of teaching failure have an impact on three dimensions of effective teaching behaviors in physical education. This finding aligns with the canonical correlation analysis results of how teaching success attributions influence effective teaching behaviors, suggesting a shared reasoning. However, there are notable differences between the correlations of teaching success and failure attributions on effective teaching behaviors. In the case of teaching success, teachers’ teaching factors are more influential, while in teaching failures, student factors hold more sway. This discrepancy is potentially related to teachers’ self-defensive psychology. Zeng et al [41] suggest that physical education teachers show more tendency to attribute teaching failures to external and unstable factors. Some scholars point out that physical education teachers often attribute successful teaching to internal, stable, and controllable factors [42]. In this light, teachers often attribute teaching success to themselves while attributing teaching failure to the external environment, a phenomenon driven by teachers’ need to uphold their self-esteem. This conclusion is consistent with many relevant studies, such as: if teachers attribute the teaching failure to themselves, they will actively take action to improve their teaching [43]. Other scholars affirm that regardless of how teachers attribute teaching failures—to external factors, students, or themselves—they will actively explore teaching principles and enhance their methods, reflecting their professional responsibility and belief in self-growth [44]. Moreover, in the realm of coaching, some scholars state that basketball coaches who attribute training results to their abilities, efforts, physical and mental states, and mutual trust with athletes, experience a higher sense of training efficacy [45,46]. In addition, physical education teachers may experience teaching deviations when they fail to quickly adapt to the constantly changing classroom dynamics [47]. The gap between idealized teaching plans and actual classroom practices is also considered a hidden factor leading to teaching failures [12]. Nonetheless, physical education teachers reflect on these deviations and subsequently revise their teaching plans [48]. The conclusions drawn by previous researchers have been validated through interviews, indicating a consistent pattern: physical education teachers improve their teaching plans in response to identified issues, embodying a commitment to professional development and teaching excellence.

Some researchers have analyzed the manifestations, causes, and improvement strategies of inappropriate teaching behaviors among physical education teachers. Their study indicates that various factors, such as school work pressure, teachers’ negative emotions, and student behavioral exhaustion, can lead to inappropriate teaching behaviors [49]. Recommended solutions primarily include reducing school burdens, enhancing emotional regulation, and maintaining a balance between criticizing and motivating students [50].

Therefore, when implementing educational reforms to address teaching issues among physical education teachers, schools should focus on how these teachers attribute teaching-related topics, minimize negative attributions, create supportive teaching environments, and improve overall teaching quality. Teachers should respond to teaching failures promptly, identify internal factors contributing to these failures, overcome teaching challenges with their efforts and abilities, and promote effective teaching behavior.

### 4.8. Representative cases

#### 4.8.1. Case study of A1.

A1, an outstanding primary school teacher, views physical education classrooms as interactive forums between teachers and students. He believes that accusing students of minor disruptive behaviors can negatively impact the learning experience. A1 attributes such incidents to unappealing classroom content, dull materials that fail to ignite enthusiasm, or inappropriate teaching methods. He emphasizes the importance of dedicating extra effort to creating engaging and stimulating content that captures students’ interest.

#### 4.8.2. Case study of A27.

A27, a junior high school teacher, suggests that when teaching ideas do not align with students’ actual needs, course content is outdated, or teaching methods are overly monotonous, it can result in a dreary classroom atmosphere. This lack of engagement leads to reduced student interest and enthusiasm.

#### 4.8.3. Case study of A33.

A33, a high school physical education teacher, posits that physical and mental health significantly impact physical education instruction. He argues that when a teacher is unwell or in a negative emotional state, they are more prone to teaching errors, such as losing focus during instruction and performing demonstrations incorrectly. Furthermore, he points out that weather conditions are another factor that greatly impacts outdoor physical education classes.

#### 4.8.4. Case study of A36.

A36, a teacher with five years of experience in primary school physical education, encountered low student evaluations during her first semester on the job. Following a discussion with the teaching and research office team leader, A36 concluded that a lack of student engagement in classroom instruction was the primary cause of her pedagogical difficulties. Inspired by this conversation, she transformed her teaching philosophy, regularly seeking feedback from students after class to refine her approach. This change led to highly effective subsequent physical education sessions. A36 believes that if students do not like the teacher, they are unlikely to appreciate his pedagogical approach.

## 5. Research limitations

The research area is confined to Henan Province, and the findings exhibit regional characteristics. To enhance the study’s applicability, future research should expand both the subjects and scope. This study employs questionnaire surveys and interviews for data collection. The former offers a broad scope of research, ensuring the acquisition of data from respondents, while the latter can address more intricate issues. However, the research is cross-sectional and lacks a longitudinal study of physical education teachers’ teaching behaviors. Additionally, it is also challenging to interpret the significance of these behaviors from cultural or life history perspectives. Therefore, it is suggested that future research should employ long-term participatory observation of physical education teaching, aiming to explain teaching beliefs, attributions of teaching success and failure, and teaching performance of physical education teachers from the perspectives of culture or life history.

The most effective approach involves employing latent growth structural equation modeling to conduct a four-year longitudinal study on the teaching behaviors of physical education teachers. The study should discuss the trajectories of changes in the teachers’ teaching beliefs, attributions of success and failure, and their teaching performance, and explore measures for improvement. This represents a valuable research endeavor, providing deeper insights and a more comprehensive understanding of physical education teaching practices over time.

## 6. Conclusion

The length of teaching experience is a primary factor influencing teaching beliefs, attribution of teaching success, and effective teaching behaviors. There is a positive correlation between physical education teaching beliefs and effective teaching behaviors. Specifically, teaching beliefs can account for 53.2% of the variance in effective teaching behaviors, and effective teaching behaviors can account for 41.5% of the total variance in teaching beliefs. Additionally, there is a closer relationship between effective teaching behaviors and capacity flexibility. Attribution of teaching success can explain 50.9% of the variance in effective teaching behaviors, and effective teaching behaviors can account for 54.2% of the total variance in attribution of teaching success. There is a positive correlation between the attribution of teaching success and effective teaching behaviors, and this correlation is particularly significant in the context of physical education teachers’ teaching factors. Similarly, the attribution of teaching failure explains 49.1% of the total variance in effective teaching behaviors, and effective teaching behaviors can account for 42.6% of the total variance in the attribution of teaching failure. Furthermore, the connection between student factors and the teaching plan is robust. It’s noteworthy that physical education teachers maintain their effective teaching behaviors even facing perceived teaching failures.

In short, the teaching beliefs and attributions of the success or failure of physical education teachers are important factors that affect effective teaching behavior. However, physical education teachers always strive to use various methods to mitigate teaching failures and enhance effective teaching behavior.

Based on the research findings, this study proposes several targeted strategies to improve physical education instruction: Physical education teachers should regularly strengthen their pedagogical beliefs to enhance their teaching effectiveness; teachers should improve their professional skills to keep pace with evolving educational practices.; teachers should fully understand their students, addressing individual needs and fostering a sense of achievement for each student; teachers should scientifically attribute their teaching successes and failures, seeking promotive or transformative teaching strategies; schools should frequently organize refresher courses or symposiums on physical education instruction; physical education teachers should regularly engage in discussions with senior teachers to promote professional growth; furthermore, teachers should manage negative emotions that affect the physical education classroom environment.

## Supporting information

S1 File Descriptive statistical analysis results. (RAR)
